# Spatiotemporal dynamics of amygdala during implicit and explicit facial emotional recognition in major depressive disorder: An MEG study

**DOI:** 10.1017/S0033291725101888

**Published:** 2025-10-24

**Authors:** Yishan Du, Yi Xia, Lingling Hua, Mohammad Ridwan Chattun, Zhongpeng Dai, Shui Tian, Wei You, Chen He, Jiayu Liu, Junling Sheng, Cong Pei, Qian Liao, YingLin Han, Hao Tang, Zhijian Yao, Qing Lu

**Affiliations:** 1 Department of Psychiatry, The Affiliated Brain Hospital of Nanjing Medical University, Nanjing, China; 2 School of Biological Sciences & Medical Engineering, Southeast University, Nanjing, China; 3 Child Development and Learning Science, Key Laboratory of Ministry of Education, Southeast University, Nanjing, China; 4 Department of Radiology, The First Affiliated Hospital of Nanjing Medical University, Nanjing, China; 5 Nanjing Brain Hospital, Medical School of Nanjing University, Nanjing, China

**Keywords:** amygdala, explicit emotion, implicit emotion, magnetoencephalography, major depressive disorder

## Abstract

**Background:**

Major depressive disorder (MDD) patients exhibit a mood-congruent emotional processing bias within the amygdala toward negative facial stimuli at both unconscious and conscious levels. Therefore, our study aimed to investigate the temporal and spatial dynamics of amygdala along with its interactions with the whole brain during implicit and explicit conditions in MDD.

**Methods:**

Thirty MDD patients and 26 healthy controls (HCs) underwent magnetoencephalography (MEG) recordings and performed implicit and explicit emotional face recognition tasks with happy, sad, and neutral facial expressions. Using the amygdala as a seed region, time frequency representations (TFR) and functional connectivity (FC) were calculated. Pearson correlation analyses measured the relationship between TFR and FC values with clinical symptoms.

**Results:**

During implicit processing, MDD patients exhibited left amygdala activation in the gamma power (60–70 Hz) before 250 ms in response to sad facial stimuli compared to HCs. In the implicit mode, there were increased FC between the right amygdala and several brain regions in the occipitoparietal lobes, as well as higher FC between the left amygdala and putamen in MDD patients. Additionally, the right amygdala was positively correlated with the severity of depression and anxiety during implicit processing.

**Conclusions:**

MDD patients had lateralized amygdala activation in response to sad facial expressions during unconscious emotional recognition of facial stimuli. Our study provided valuable insights into the spatiotemporal dynamics of facial emotional recognition associated with depressive and anxiety-related cognitive bias during implicit and explicit processing.

## Introduction

Major depressive disorder (MDD) is a multidimensional disorder that is accompanied by biased recognition of facial expressions. MDD patients are prone to focus on sustained negative rather than positive stimuli, interpret ambiguous events negatively, and selectively recall negative memories (Duyser et al., 2020). Mood-congruent cognitive biases in depressed mood are usually fueled by anxiety (Blanco, Boemo, & Sanchez-Lopez, 2021), depressogenic experiences, and negative thinking (Jiang, 2024). Furthermore, negativity bias might contribute to the onset of depression (Gray, Douglas, & Porter, 2021), and is closely related to depression severity (Lee, Mathews, Shergill, & Yiend, 2016).

While the processing of emotional information is complex, it is regarded as a continuum ranging from an implicit/unconscious level to an explicit/conscious level (Celeghin, Mazzoni, & Mattavelli, 2020). It is widely acknowledged that the amygdala is a central structure in the limbic emotion processing circuit. The amygdala primarily receives information through dual pathways: (1) a slow cortical pathway which transmits emotional information to the amygdala through the visual cortices and engages in higher order cognitive processes to enable conscious awareness (Simic et al., 2021), and (2) a fast subcortical retino–colliculo–pulvinar–amygdala route, which mediates automatic, unconscious emotional processing and encodes negative emotion without conscious supervision (Kragel et al., 2021). Accumulating evidence suggests that implicit and explicit emotion processing have distinct neural substrates across different time scales (Mazzoni, Celeghin, & Mattavelli, 2024; Tao et al., 2022). Although visual information from the amygdala takes over 20 ms to reach primary and secondary visual cortex, V1 and V2 (Carretié, Yadav, & Méndez-Bértolo, 2021), the emotion-related response of the visual cortex is reported to occur prior to 100 ms from stimulus onset (Mendez-Bertolo et al., 2016). Within the first 100–200 ms after facial expression stimulation, the brain unconsciously engages in perceptual analysis, meaning extraction, cognitive processing, and action organization (Brunet, 2023). Facial expressions are typically recognized and differentiated within the 200–250 ms after presentation (Calvo & Nummenmaa, 2011; Lin & Liang, 2023). After the stimulus, the emergence of consciousness occurs approximately after 300–400 ms (Knyazev, Slobodskoj-Plusnin, & Bocharov, 2010). A magnetoencephalography (MEG) study on unconscious emotional face processing supported the concept of a rapid subcortical pathway and a slower cortical pathway by revealing that early amygdala activations (40–140 ms post-stimulus) were followed by slower responses (280–410 ms) in the frontoparietal cortex (Luo et al., 2010). Previous studies have suggested that MDD patients displayed an attentional bias to sad faces, even when the stimulus is presented for >1000 ms (Krause, Linardatos, Fresco, & Moore, 2021).

A number of evidence suggest that individuals with MDD exhibit enhanced sensitivity to negative emotional stimuli during unconscious processing. Functional magnetic resonance imaging (MRI) studies (Li, Li, Hu, Yang, & Luo, 2024; Nawaz, Shah, & Ali, 2023) have shown that, compared to healthy controls, MDD patients displayed a hyperactivation of the amygdala in response to masked sad faces and diminished responses to masked happy faces. Electrophysiological findings (Dellert et al., 2021; Qiu, Lei, Becker, & Pegna, 2022) further supported this heightened sensitivity, with increased amplitudes of P100/M100 and N170/M170 components and shortened latencies of P300/M300 during unconscious emotional face processing. At the neural oscillatory level, abnormal theta and gamma band activity has been observed in response to masked negative stimuli (Chen et al., 2025; Gilbert, Galiano, Nugent, & Zarate, 2021), and reduced alpha–gamma cross-frequency coupling has been associated with poorer treatment outcomes (Dai et al., 2022). While some studies in healthy individuals have reported stronger bilateral amygdala responses to masked happy faces than to sad ones (Juruena et al., 2010), this pattern appears to be reversed or disrupted in MDD. Specifically, decreased amygdala responses to masked happy faces have been linked to greater depression severity (Sergerie, Chochol, & Armony, 2008), and higher levels of anhedonia (Stuhrmann et al., 2013). Altogether, these findings highlight a negativity bias in MDD that emerges at an early, automatic stage of emotional processing, characterized by amygdala hyperactivity to unconsciously perceived negative emotional cues.

MEG has an excellent spatiotemporal resolution and is a favorable tool for investigating facial emotion recognition since it is able to provide real-time insights about high-frequency bands, such as gamma, which is crucial in cognitive, perceptual, and emotional processes. It has been demonstrated that the activities of neural frequency bands, including theta, alpha, beta, and gamma, played distinct roles in various stages of emotion processing. Recent studies have demonstrated that MEG could reliably capture amygdala activity and differentiate it from hippocampal signals using advanced source reconstruction techniques (Attal et al., 2007; Ishizaki et al., 2025; Styliadis, Ioannides, Bamidis, & Papadelis, 2014). In addition, we further enhanced the accuracy of source localization by acquiring individual structural MRI scans to construct subject-specific individualized head models. The aim of this study was to explore the neural mechanisms underlying implicit and explicit processing of emotional facial expressions in MDD patients. We hypothesized that cognitive biases exist in depressed patients during both explicit and implicit processing, and that differential amygdala activation patterns would emerge between the two emotion processing conditions.

## Methods and materials

### Participants

A total of 56 participants, including 30 MDD patients from the inpatient departments of Nanjing Brain Hospital and 26 healthy controls (HCs) from the local community, were recruited between January 2022 and July 2023. All participants were right-handed, aged 18–60 years, completed a minimum of nine years of education, and had normal or corrected-to-normal visual acuity. Patients were diagnosed with MDD by two senior psychiatrists according to the Diagnostic and Statistical Manual of Mental Disorders, 5th edition (DSM-5) (American Psychiatric Association, 2013). Two senior psychiatrists who underwent standardized training used the 17-item Hamilton Depression Rating Scale (HAMD-17) (Hamilton, 1960) and Hamilton Anxiety Rating Scale (HAM-A) (Hamilton, 1959) to assess the severity of depression and anxiety in MDD patients.

### Inclusion and exclusion criteria

The inclusion criteria for MDD patients were as follows: (1) Diagnosed with MDD based on DSM-5 criteria by two senior psychiatrists; (2) HAMD-17 scores >17; (3) no history of other Axis-I psychiatric disorders; (4) no electroconvulsive therapy and psychotherapy within the last six months. MDD patients were excluded if they exhibited any of the following conditions: (1) presence of major medical or neurological disorders; (2) diagnosis of dementia or significant cognitive impairment (Mini-Mental State Examination (Folstein, Robins, & Helzer, 1983) score < 24); (3) a history of substance dependence or abuse within the past year or throughout their lifetime; (4) pregnant or breastfeeding; (5) contraindication for MEG such as a pacemaker, hearing aid, or dental implants. For HCs, exclusion criteria comprised: (1) neurological or psychiatric conditions; (2) a family history of major psychiatric disorders in first-degree relatives; and (3) additional exclusion criteria were similar with MDD patients.

### Experimental design

**Figure 1.**
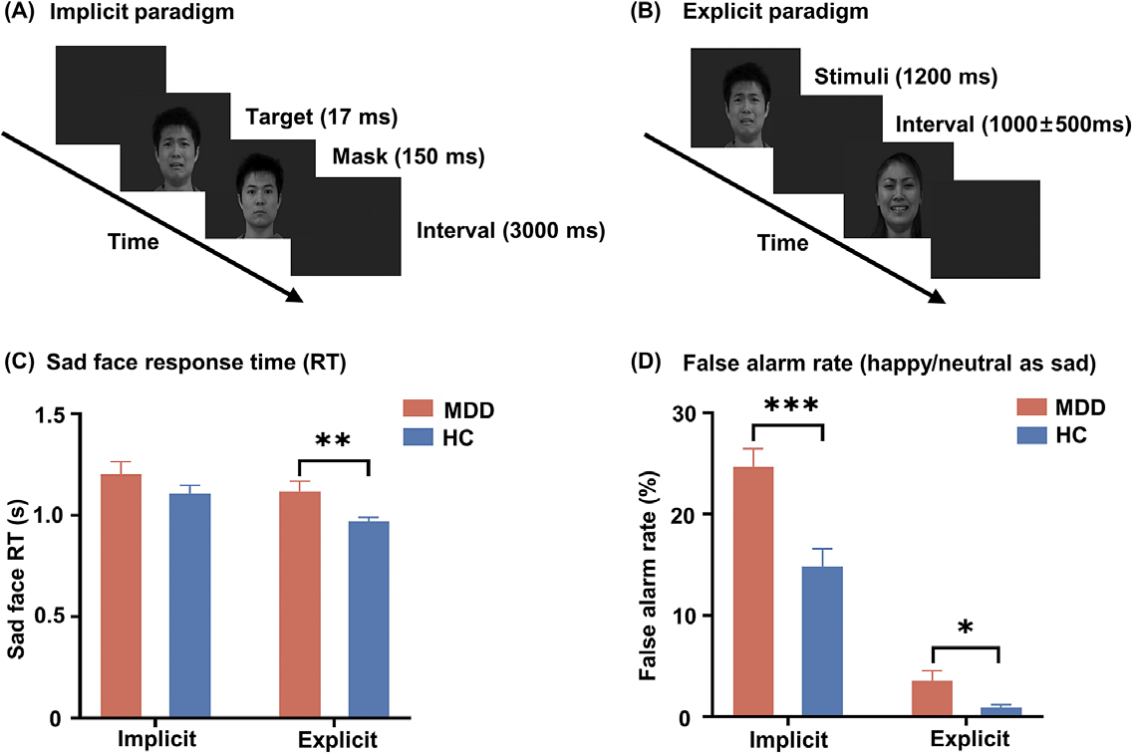
(A) Implicit paradigm: backward masking task. (B) Explicit emotional recognition paradigm. (C) Sad face response time (RT): comparison of response times to sad faces between the two groups was conducted under each of the two paradigms. (D) False alarm rate (happy/neutral as sad): proportion of happy and neutral faces incorrectly identified as sad. Error bars represent the standard error of the mean. **P < 0.01; ***P < 0.001.

In both explicit and implicit tasks, a total of 24 grayscale normalized facial images were selected from the Chinese Facial Expression Video System (Du et al., 2007). These images featured eight individuals, including four distinct males and four different females, with sad, happy, and neutral expressions. The emotional paradigm was executed using the Brain-X software (Developed by Cincinnati Children’s Hospital, USA) that presented stimuli in a pseudo-randomized sequence. The response accuracy and reaction times (RT) to each stimulus presentation were documented for each participant. The experimental paradigm was shown in Figure 1A and B.

For both paradigms, participants were tasked to quickly and accurately identify facial expressions by pressing the button corresponding to each expression with the right hand. They underwent a training phase before the start of MEG recordings. In the implicit task, sad, happy, and neutral faces were used as target stimuli, followed by a backward masking procedure with neutral faces. In order to eliminate any potential confusion between neutral faces used as targets and masks, neutral target faces were displayed as a mirror-inverted version of the neutral mask face. The target face was presented for 33 ms, followed by a masked face displayed for 150 ms. This duration is commonly used in backward masking paradigms to minimize conscious awareness of the target stimulus (Gunther et al., 2017; Pessoa, Japee, & Ungerleider, 2005). There were 40 trials for each expression; each trial was separated by a 3000-ms interval. The task was divided into two sections, each containing 120 stimuli and lasting approximately 6.3 min. In the explicit task, sad, happy, and neutral expressions were displayed for 1.2 s with random inter-stimulus intervals of 1.5, 2, or 2.5 s. The session included a total of 120 stimuli, with 40 of each expression, and the total duration was approximately 5.4 minutes. There was a 5-min break between two tasks in order to minimize the potential legacy effects from implicit tasks to explicit tasks.

### MEG and MRI data acquisition

All participants underwent MEG recordings in a magnetically shielded room at the MEG Laboratory of Nanjing Brain Hospital. MEG data were continuously recorded at a sampling rate of 1200 Hz using a whole-head 275-channel axial gradiometer system (CTF Omega 2000, Canada). During the recordings, participants were instructed to lie in a supine position and were required to satisfy the criteria of maximum head motion in translation <5 mm. If any participant’s head displacement exceeded 5 mm, the corresponding data were removed, and the participant was asked to repeat the task while being reminded to keep their head still. Three fiducial markers were placed at the left and right canals and the nasion to ensure MEG and offline MRI co-registration.

Following the MEG scan, all participants underwent an MRI T1 scan for precise head localization. The structural T1 images were acquired via a Siemens Verio3T MRI system with a high-resolution, T1-weighted, and 3D gradient-echo pulse sequence. The specific scanning parameters were as follows: repetition time (TR) = 1900 ms, echo time (TE) = 2.48 ms, flip angle (FA) = 9°, slice thickness = 1 mm, the number of slices = 176, acquisition voxel size = 1 × 1 × 1 mm^3^, matrix = 256 × 256, and field of view (FOV) = 250 × 250 mm^2^.

### MEG preprocessing and source reconstruction

MEG data preprocessing was performed using the Fieldtrip toolbox of MATLAB (fieldtrip.fcdonders.nl; version 20231025). Continuous data were down-sampled to 600 Hz. Power line interference within the 49–51 Hz range was removed using a band-stop filter. Stimuli were segmented into epochs spanning from −1000 to 1000 ms with respect to stimulus onset. Trials and channels with excessive variance were also eliminated. Next, independent component analysis (ICA) was applied to remove interference caused by eye blinks, heartbeats, and muscle movements through visual inspection.

The MRI data were converted from the original DICOM format to 3D NifTI format by the cm2nii tool in the MRIcron software (http://www.mricro.com;v1.0.20190902). Thereafter, an anatomically-based volume conduction model of the head was generated. The MEG data that were segmented into epochs were then mapped onto a grid within the Montreal Neurological Institute (MNI) space, with a grid step size of 6 mm and utilizing the linearly constrained minimum variance (LCMV) beamforming approach. Spatial filters were derived from the lead fields for each grid point and the covariance matrix of the signal data. Subsequently, the spatial orientations of each epoch were adjusted to maximize variance. Finally, source activity was determined by multiplying the spatial filters with the sensor-level time series across the entire frequency range. The time series of virtual voxels were computed using the Automated Anatomical Labeling 90 (AAL 90) template atlas.

### Time–frequency representation analysis

TFRs were calculated on the time series of the left and right amygdala based on their central coordinates. In a low-frequency range spanning from 4 to 30 Hz, an adaptive time window of four cycles per frequency (i.e. ΔT = 4 /f) multiplied by a Hanning taper was applied. For a high-frequency range of 60–90 Hz, three orthogonal Slepian tapers with a 50-ms fixed sliding window were employed. Then, the power at each time point was divided by the average power during the baseline period (−400 to −200 ms) to obtain the percentage change in power. The time period of interest for statistics was 50–500 ms. Differences between implicit and explicit emotion paradigms were explored within each group as well as comparisons between groups.

### Functional connectivity analysis

In order to explore the potential interactions between the amygdala and other brain regions during sad emotion processing, the amygdala was selected as the seed point to assess its functional connectivity (FC) with 89 other brain regions during specific time and frequency intervals derived from TFR results in both MDD and HCs groups. Firstly, phase information was extracted from the preprocessed data for each epoch and channel within the chosen frequency band. Subsequently, paired phase locking value (PLV) across time windows was calculated between the amygdala and other brain regions. An FC matrix was then constructed, with each element representing the PLV value between two brain regions. The resulting 89 connections were averaged to yield the mean FC of the entire brain with the amygdala as the central node.

### Statistical analysis

Demographic and clinical characteristics were compared between the MDD and HCs groups using IBM SPSS 25.0. For continuous variables, *t*-tests or non-parametric tests were employed while chi-square tests were utilized for categorical variables. Age and sex were included as covariates in all group-level statistical analyses to avoid potential confounding effects. To account for potential medication effects, a composite Medication Load Index was calculated for each patient following the guidelines of the Modified Antidepressant Treatment History Form (Sackeim, 2001). This method standardizes antidepressant exposure across different drug classes by categorizing each treatment into ordinal levels (0–5) based on the specific agent, dose, and treatment duration. For patients receiving combination therapy, the scores for each antidepressant were summed to yield a total MLI score. The final MLI scores were then included as covariates in all subsequent statistical analyses to control for the influence of medication exposure. The signal discrimination index (d′) was used to measure the effectiveness of the backward masking task design according to signal detection theory. One-sample *t*-tests were conducted to assess the differences between each sample and d’. A multivariate analysis of variance (MANOVA) was conducted to explore the interactive effects on RT, with group (MDD versus HCs) serving as a between-subjects factor, and task (implicit versus explicit) and emotion (sad, happy, neutral) as within-subjects factors. Furthermore, Independent samples *t*-tests were used to examine the RT and the false alarm rate of sad faces between MDD and HCs groups. One-way analysis of variance was utilized to analyze the mean FC values between the two groups. The statistical significance level was set as *p* < 0.05 (two-tailed).

In the analysis of TFR data, we used a non-parametric cluster-based permutation *t*-test to identify statistically significant differences across different time points and frequencies. This method effectively controls the false-positive rate of multiple comparisons by leveraging the spatial and temporal structure of the data, generating a null hypothesis distribution, controlling the false positive rate, and using cluster-level statistics. The specific methods are as follows: for each comparison, a *t*-test is performed at each time–frequency point to identify preliminary significant effects. Contiguous significant points are grouped into clusters, and a cluster-level test statistic is computed as the sum of the *t*-values within each cluster. Group labels underwent random shuffling 5000 times in order to obtain a stable cluster distribution. During each shuffling, the clusters with the highest *t*-values were utilized to generate the reference distribution. Any observed cluster statistic values surpassing the 95th percentile of the reference distribution was regarded as statistically significant (*P* < 0.05, cluster-level corrected). Independent samples *t*-test were used to compare the differences in PLV values between the amygdala and other brain regions across different time windows and frequency bands between the MDD and HC groups, and the false-discovery rate (FDR) correction was used to account for multiple comparisons.

Pearson correlation analyses were performed to examine the associations between clinical characteristics (HAMD-17 and HAMA scores) and neurophysiological measures, including TFRs of the amygdala and FCs. The FDR correction was applied across all correlation tests to account for multiple comparisons.

## Results

### Demographic and clinical characteristics

A total of 30 MDD patients and 26 HCs were ultimately included in the final analysis. Among them, two MDD patients exceeded the 5-mm threshold for head movement during their initial MEG recording and were rescanned to ensure data quality. The demographic and clinical assessments of all participants are illustrated in Table 1. There were no significant differences in age and sex between the two groups.

**Table 1.** Demographic and clinical data of the participants

Characteristics	MDD (n = 30)	HC (n = 26)	*P*-value
Age (years)	25.90 ± 9.20	25.88 ± 4.48	0.994
Gender (F/M)	20/10	17/9	0.920
Family history, N (%)	9 (30%)	–	–
Illness duration (months)	60.83 ± 56.38	–	–
Age of onset (years)	20.90 ± 7.44	–	–
Number of episodes	1.46 ± 0.51	–	–
HAMD–17	21.27 ± 4.39	–	–
HAMA	19.77 ± 7.01	–	–
Number of patients under antidepressant medication (yes/no)	22/8	–	
SSRIs	14	–	–
SNRIs	6	–	–
SSRIs + NaSSA	2	–	–
Medication load index	1.07 ± 0.25	–	–

*Note*: Data are presented as mean ± standard deviation. F, female; HAMA, the Hamilton Anxiety Rating Scale; HAMD-17, the 17-item Hamilton Rating Scale for Depression; HC, healthy control; M, male; MDD, major depressive disorder.

### Behavioral task performance

Following the implicit emotion examination, all subjects reported an inability to discern any of the emotional faces that were displayed briefly, despite being informed of their presence. One-sample *t*-test revealed that there were no statistically significant differences between d′ and zero in the implicit condition for all subjects (*t* = 0.297, *P* > 0.05), suggesting that the observed neurobiological response occurred at an unconscious level, in the absence of conscious awareness of the emotional stimulus.

The results of MANOVA indicated a significant main effect of task (F (1,54) = 10.940, *P* = 0.001, η^2^ = 0.042), indicating that participants responded significantly faster (1.038 ± 0.281 s) during the implicit task compared to the explicit task (1.228 ± 0.511 s). The main effect of group was not statistically significant (F (1,54) = 3.344, *P* = 0.069), although MDD patients showed a tendency toward slower RTs compared to HCs. However, the Group × Task × Emotion interaction was not statistically significant (F (2,54) = 2.062, *P* = 0.130, η^2^ = 0.016), indicating that the effects of emotion and task on RT did not significantly differ across groups.

Although the interaction effect at the time of response was not significant, we further explored between-group differences in RT of the sad face across task conditions. In the implicit emotion task, there was no significant difference in RT to sad facial stimuli between the MDD group (1.204 ± 0.331 s) and the HCs (1.106 ± 0.216 s). However, in the explicit emotion task, MDD patients exhibited significantly slower RT to sad faces (1.119 ± 0.273 s) than HCs (0.969 ± 0.117 s, *P* = 0.009) (see Figure 1C). In addition, MDD patients were more likely to classify neutral or happy facial expressions as sad faces in both implicit (*P* = 0.011) and explicit (*P* = 0.017) emotion processing (e.g. Figure 1D).

### TFR of amygdala in implicit and explicit tasks

**Figure 2.**
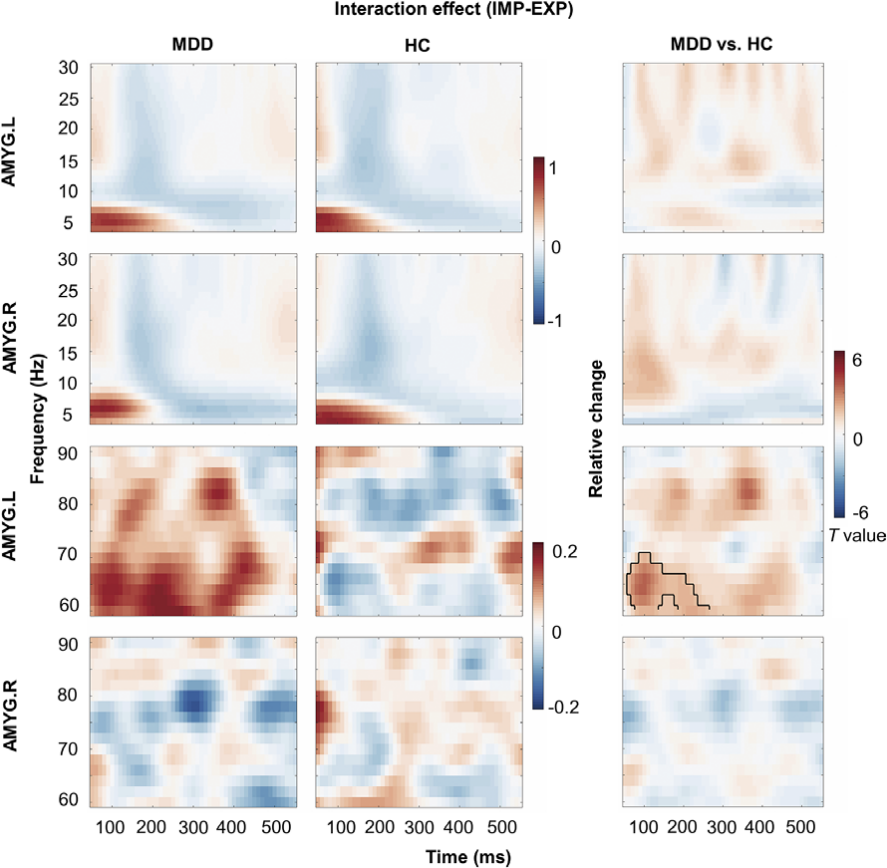
Time–frequency representation (TFR) differences of the amygdala in different emotional processing modes. Interaction effect between groups and emotion processing modes. The colors represent power changes; red indicates an increase in power, while blue denotes a reduction in power.

Non-parametric cluster-based permutation tests revealed significant inter-group differences in both experiments (see Supplementary Material, Figure S1). Figure 2 highlights the interaction effects between groups and experimental paradigms. The comparative analysis of the two paradigms revealed that MDD patients exhibited left amygdala activation within the gamma frequency band (60–70 Hz), which was observed before 250 ms compared to the HC group (cluster, *P* = 0.018) after facial stimuli.

**Figure 3.**
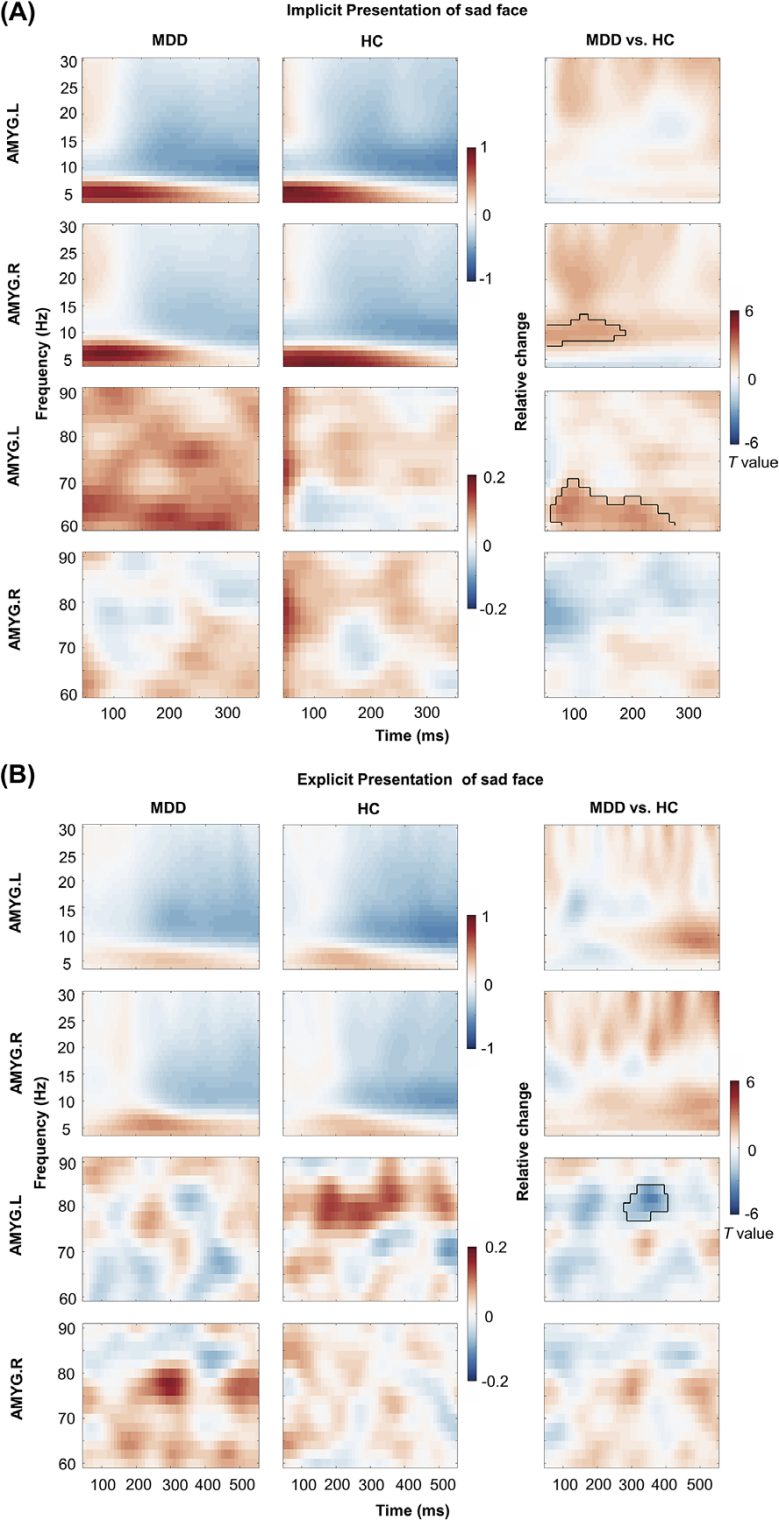
Inter-group comparison of time–frequency representation (TFR) in bilateral amygdala during different emotional processing modes. (A) TFR and statistical comparison of bilateral amygdala to sad face between MDD group and HC group in the implicit condition. (B) TFR and statistical comparison of bilateral amygdala to sad face between MDD group and HC group in the explicit condition. The colors represent power changes; red shows an increase in power, while blue signifies a reduction in power.

Figure 3A and B depicts the comparative TFR of bilateral amygdala activity to a sad face between the MDD and HC groups under implicit and explicit conditions, respectively. In the implicit task, the MDD group showed enhanced alpha power in the right amygdala within the 50–200 ms time frame (cluster, *P* = 0.049; not surviving FDR correction) and a significant increase in gamma power of the left amygdala around 50–250 ms following stimuli (cluster, *P* = 0.006; survived FDR correction) compared to HC group. In the explicit experiment, the MDD group exhibited a decrease in the left amygdala gamma power during the 300–400 ms post-sad facial expression presentation (cluster, *P* = 0.045; not surviving FDR correction) in contrast to the HC group.

To determine whether the observed amygdala activation in MDD was specifically driven by negative emotion rather than neutral face processing, we conducted additional within- and between-group comparisons under the implicit condition. Within the MDD group, compared to neutral face, sad faces elicited significantly stronger right amygdala responses in alpha and beta band (100–250 ms) (*P*_cluster_ < 0.05), and greater left amygdala activity in gamma band (50–250 ms) (*P*_cluster_ < 0.05) (see Supplementary Figure S2). Moreover, between-group comparisons for neutral faces revealed no significant differences (see Supplementary Figure S3).

### Functional connectivity of the amygdala

**Figure 4.**
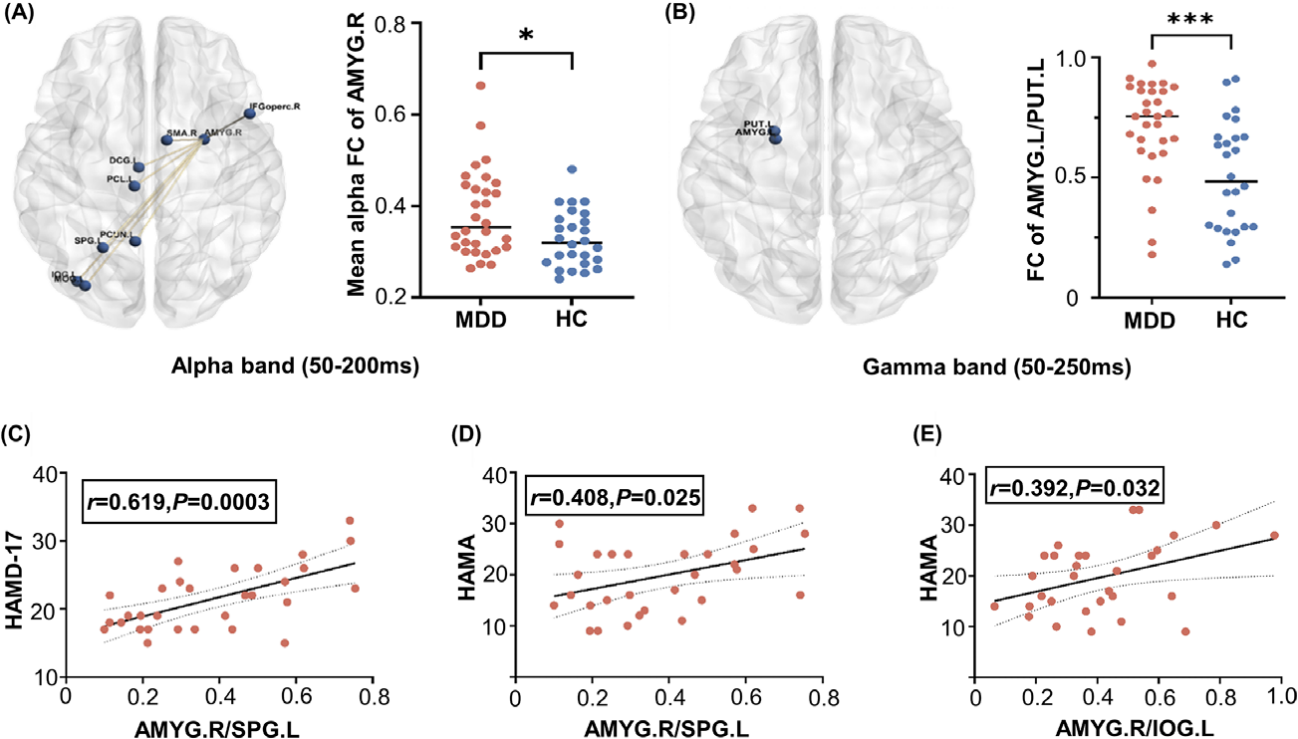
(A) Group differences in functional connectivity (FC) in the alpha band (50–200 ms) between MDD patients and HCs, with corresponding mean FC comparisons (*P < 0.05). (B) Significant FC difference in the gamma band (50–250 ms) between the left amygdala (AMYG.L) and the left putamen (PUT.L) in MDD patients compared to HCs (***P < 0.001). (C) Positive correlation between FC of the right amygdala (AMYG.R) and the left superior parietal gyrus (SPG.L) with HAMD-17 scores. (D) Positive correlation between FC of AMYG.R and SPG.L with HAMA scores. (E) Positive correlation between FC of AMYG.R and the left inferior occipital gyrus (IOG.L) with HAMA scores.

In the implicit task, there were significant differences in the FC between the amygdala and other brain regions. MDD patients demonstrated higher FC in the right amygdala in the alpha frequency range between 50 and 200 ms (*P* = 0.014, survived after FDR) (see Figure 4A and B). This hyperconnectivity encompassed various brain regions, including the right amygdala (AMYG.R) with the left inferior occipital gyrus (IOG.L) and left middle occipital gyrus (MOG.L); AMYG.R with the left paracentral lobule (PCL.L), left superior parietal gyrus (SPG.L) and left precuneus (PCUN.L); AMYG.R with the left median cingulate and paracingulate gyrus (DCG.L); AMYG.R and the right inferior frontal gyrus (IFGoperc.R); and AMYG.R with the right supplementary motor area (SMA.R). Additionally, an increased FC was observed between the left amygdala (AMYG.L) and the left putamen (PUT.L) in the gamma frequency range between 50 and 250 ms (*P* < 0.001, survived after FDR) (see Figure 4C and D). However, during the explicit experiment, no significant group difference were observed in the FC between the amygdala and other brain regions.

### Clinical correlation analysis

In the implicit emotion task, correlation analyses revealed that the FC between the AMYG.R and SPG.L was positively correlated with both HAMA scores (*r* = 0.408, *P* = 0.025, survived after FDR) and HAMD-17 scores (*r* = 0.619, *P* < 0.001, survived after FDR), as shown in Figure 4C and D. In addition, the FC between AMYG.R and IOG.L was positively associated with HAMA scores (*r* = 0.392, *P* = 0.032, survived after FDR), as shown in Figure 4E. Non-significant correlations between FCs and clinical variables are reported in Supplementary Table S2.

## Discussion

Behavioral task performance revealed that MDD patients interpreted neutral and happy faces as a sad expression in emotion recognition under both implicit and explicit conditions, implying the presence of a cognitive bias in MDD. This result is consistent with previous studies, which showed that MDD patients had a tendency to perceive higher levels of negative emotions and diminished positive emotions (Krause et al., 2021; Maniglio et al., 2014). MDD patients also had slower reactions to sad facial expressions compared to HCs in the explicit emotion experiment, which is in accordance with the findings of prior studies. These findings might be attributed to emotional recognition disturbances and cognitive biases in individuals with MDD.

In the present study, group comparison revealed a significant gamma activation (50–250 ms post-stimuli) in the left amygdala during the implicit task relative to the explicit task in MDD patients compared to HCs. A systematic review revealed that the left amygdala was consistently more often activated in comparison to the right amygdala (Baas, Aleman, & Kahn, 2004). In line with our findings, there was a higher response to sad facial stimuli in the left amygdala under implicit conditions, suggesting that there was a preferential engagement of the left amygdala during emotionally valenced stimuli (Blair, Morris, Frith, Perrett, & Dolan, 1999). These findings support the ideology that the left amygdala plays a more comprehensive role in processing emotional stimuli. The amygdala generates gamma oscillations in response to emotionally aversive stimuli, which is achieved through the linkage of perceptual visual stimuli with their emotional significance (Oya, Kawasaki, Howard 3rd, & Adolphs, 2002). In an implicit emotion experiment, gamma activation in the left amygdala peaked at ~100 ms in response to negative facial expressions, which is consistent with our findings (Liu, Chen, Hsieh, & Chen, 2015). The early gamma activation in the left amygdala identified in the current study might represent an initial heightened response to negative stimuli, and the subsequent increase could reflect disturbances in sustained emotion processing or cognitive appraisals in MDD. These findings indicate the link between MDD and higher sensitivity to emotionally salient cues in the processing of facial emotional recognition.

During implicit processing, MDD patients exhibited increased FC between the AMYG.R and multiple cortical regions in the alpha band, including the IOG.L, MOG.L, DCG.L, PCUN.L, PCL.L, IFGoperc.R, SPG.L, and SMA.R. These widespread alpha-band connections suggest that there is enhanced large-scale network coordination involving brain regions responsible for visual processing, attention, and top-down cognitive regulation (Liu, Bengson, Huang, Mangun, & Ding, 2016; Wang, Rajagovindan, Han, & Ding, 2016). In contrast, we observed gamma band hyperconnectivity between the AMYG.L and the PUT.L, highlighting a specific engagement of the dorsal striatal–amygdala circuit during early unconscious recognition of negative emotional expressions. Gamma band activity is thought to reflect rapid, localized processing of emotionally salient stimuli, consistent with a bottom-up mechanism (Martini et al., 2012; Richter, Thompson, Bosman, & Fries, 2017). Together, these results underscore the dual role of the amygdala in implicit emotional processing in MDD, engaging both long range integrative alpha band pathways and local evaluative gamma band circuits. Our findings are consistent with prior research which showed significantly higher FC between the amygdala and these brain regions during negative emotional facial stimulation in MDD (Gou et al., 2023; Zhang et al., 2020). Given that both cortical and subcortical parallel pathways are implicated in the visuospatial emotional processing, our findings suggested that the amygdala serves dual-frequency roles in unconscious emotional processing in MDD. Furthermore, bilateral amygdala involvement was observed, with right amygdala alpha band connectivity supporting broader visual–emotional integration, whereas left amygdala gamma band activity may serve as a localized marker of the emotional valence of visual inputs. This frequency-based asymmetry pattern provides insights into the pathophysiological basis of emotional bias and hypersensitivity in MDD.

Correlation analyses revealed significant positive associations: (1) between the AMYG.R and SPG.L regions and the severity of both anxiety and depression, and (2) between the AMYG.R and IOG.L regions and the severity of anxiety. Several studies have shown that aberrant FC in the AMYG, SPG, and IOG were associated with anxiety conditions and the execution of facial expressions during the recognition of negative affect (Berluti, Ploe, & Marsh, 2023; Yang et al., 2019). In healthy individuals, anxiety has been found to be positively correlated with IOG.L activity and negatively correlated with FC between IOG.L and SPG.L, indicating that the frontoparietal network plays a key role in anxiety regulation (Li et al., 2020). Joormann and Stanton (2016) reported that individuals with increased depression severity and anxiety displayed cognitive biases in response to emotional stimuli. Notably, the FC of AMYG.R and SPG.L was significantly associated with the severity of both anxiety and depression. The SPG, a critical node of the dorsal attention network, is primarily engaged in top-down modulation of attention and spatial orientation (Dixon et al., 2018). Altered FC between the SPG and amygdala might reflect maladaptive attentional allocation toward emotionally salient or threatening stimuli (Mukherjee et al., 2012). This disruption could lead to difficulty in disengaging from negative stimuli and prolonged attentional capture by emotionally salient information in MDD patients, which are hallmarks of negative cognitive bias in both anxiety and depression. Dysregulation of negative affect processing is a central component in the development and maintenance of both anxiety and depressive disorders (Barlow, Allen, & Choate, 2016). These conditions also share overlapping neurocognitive features, including heightened negative affect and increased sensitivity to negative emotional stimuli. Taken together, our findings support the notion that anxiety and depressive symptoms are linked to increased cognitive biases and disrupted information processing within affective-attentional networks.

This study has several limitations. Firstly, the sample size is relatively small, which may limit the statistical power of our findings, and future studies should expand the sample size to verify the robustness and generalizability of the observed effects. Secondly, although we quantified drug exposure using a standardized drug load index, drug treatment duration was not systematically recorded, which could potentially affect the neural activity of the amygdala. Thirdly, we did not collect post-task feedback from participants regarding the facial stimuli, and thus cannot confirm whether the emotional valence of the stimuli was perceived as intended. Fourthly, due to the limited spatial resolution of MEG, the source-localized signals attributed to the amygdala may partially overlap with those from adjacent structures such as the hippocampus, and therefore need to be interpreted with caution. Lastly, although the FDR correction was used when performing between-group comparisons and correlation analyses, the statistical power is still limited. Therefore, these results should be interpreted with caution, and independent replications in larger samples should be performed to confirm their robustness.

In summary, this study revealed that MDD patients exhibited higher gamma power in the left amygdala during implicit processing in contrast to HCs. The observed FC alterations suggested that negative biases in MDD are supported by an extended network of brain regions connected to the amygdala which participate in the evaluation of stimulus salience. Moreover, the observed positive correlations highlighted the interconnectedness of negative affect with cognitive bias in MDD presenting with anxiety symptoms. Our findings emphasized the lateralized engagement of the amygdala in conscious and unconscious emotion processing which implied a heightened sensitivity to emotionally salient cues in MDD patients, potentially influencing the processing of emotional information. These insights contributed to a deeper understanding of the underlying neural mechanisms of emotional processing in MDD, offering potential implications for therapeutic interventions targeting emotional regulation disturbances in MDD.

## Supporting information

Du et al. supplementary material Du et al. supplementary material

## Data Availability

Data shall be made available upon reasonable request to the corresponding author.

## Supplementary material

The supplementary material for this article can be accessed at http://doi.org/10.1017/S0033291725101888.
