# Supplementary material for: Spatiotemporal dynamics of amygdala during implicit and explicit facial emotional recognition in major depressive disorder: An MEG study
Source: Psychol Med. 2025 Oct 24;55:e318. doi: 10.1017/S0033291725101888 (PMC12558623; doi:10.1017/S0033291725101888)
Supplement: Du et al. supplementary material [file S0033291725101888sup001.zip › Supplementary Material-2.docx]

**Supplementary Material**

1. **Comparison of amygdala TFR in implicit and explicit tasks of sad face**

Non-parametric cluster-based permutation tests revealed significant inter-group differences in both implicit and explicit experiments of sad face. Figure S1 (A) and (B) illustrates the distinct patterns in TFR of amygdala responses during sad facial emotional processing in MDD patients and HCs when the two paradigms were compared. In MDD patients, there was an early amygdala response in both alpha (8-12Hz) and beta power at 100-250ms after stimuli (cluster, *P <* 0.001). In contrast, amygdala activation of the theta power was markedly delayed which occurred between 250 to 500ms post-stimuli (cluster, *P*<0.001). In addition, MDD patients exhibited two distinct surges in the gamma frequency band (60-70Hz) of the left amygdala, with the first one occurring at 50-150ms (cluster, *P*=0.033) and the second one at 150-300ms (cluster, *P*=0.049) following facial presentations. HCs exhibited comparable amygdala reactions in alpha and beta power (cluster, P<0.001) but did not demonstrate any gamma power activation in the amygdala similar to MDD patients. Figure 2 (C) highlights the interaction effects between groups and experimental paradigms. Significant paradigm-dependent differences in the left amygdala activation within the gamma frequency band (60-70 Hz) were observed before 250ms between the MDD and the HC groups (*P*=0.018) after facial stimuli.

1. **TRF Analysis of Amygdala Responses to Neutral Faces in MDD during implicit task**

In order to validate that amygdala activation in MDD was specifically driven by the sad emotion effect rather than neutral face processing, we conducted additional analyses under the implicit condition. First, within the MDD group, compared to neutral faces, sad faces elicited significantly greater alpha and beta activity in the right amygdala (100 - 250 ms) (*P*_cluster_ < 0.05), and enhanced gamma band activity in the left amygdala (50 - 250 ms) (*P*_cluster_ < 0.05) (see

Supplementary Figure S2); Furthermore, between-group comparisons for neutral faces revealed no significant differences in amygdala activity between MDD and HC groups, suggesting that the observed group differences were not driven by neutral face processing (see Supplementary Figure S3).

These findings collectively supported the interpretation that the enhanced amygdala response in MDD was specifically triggered by negative emotional stimuli, rather than neutral face perception. This is consistent with the negative mood bias mechanism in MDD and further supports a key role for the amygdala in emotion processing. We have clarified these analyses in the Supplementary Materials.

**3.Table S1:**  **Scoring Criteria for Medication Load Index Based on the Modified** **ATHF**

| Medication Class | Drug Name | Dose Range | Score |
| --- | --- | --- | --- |
| SSRI | Fluoxetine (Prozac), Citalopram (Celexa) | drug < 4 wks or 4 wks or more and dosage 1-9 mg/day | 1 |
|  |  | 4 wks or more and dosage 10-19 mg/day | 2 |
|  |  | 4 wks or more and dosage 20-39 mg/day | 3 |
|  |  | 4 wks or more and dosage 40 mg/day | 4 |
|  | Fluvoxamine (Luvox) | drug < 4 wks or drug < 100 mg/day | 1 |
|  |  | 4 wks or more and dosage 100-199 mg/day | 2 |
|  |  | 4 wks or more and dosage 200-299 mg/day | 3 |
|  |  | 4 wks or more and dosage 300 mg/day or greater | 4 |
|  | Paroxetine (Paxil/ Seroxat) | drug < 4 wks or 4 wks or more and dosage | 1 |
|  |  | 4 wks or more and dosage 10-19 mg/day | 2 |
|  |  | 4 wks or more and dosage 20-29 mg/day | 3 |
|  |  | 4 wks or more and dosage 30 mg/day | 4 |
|  | Sertraline (Zoloft) | drug <4 wks or 4 wks or more and dosage < 50 mg/day | 1 |
|  |  | 4 wks or more and dosage 50-99 mg/day | 2 |
|  |  | 4 wks or more and dosage 100-199 mg/day | 3 |
|  |  | 4 wks or more and dosage 200 mg/day | 4 |
|  | Citalopram (Celexa) | drug < 4 wks or 4 wks or more and dosage 1-9 mg/day | 1 |
|  |  | 4 wks or more and dosage 10-19 mg/day | 2 |
|  |  | 4 wks or more and dosage 20-39 mg/day | 3 |
|  |  | 4 wks or more and dosage 40 mg/day | 4 |
| SNRI | Duloxetine (Cymbalta) | drug < 4 wks or 4 wks or more and dosage < 30 mg/day | 1 |
|  |  | 4 wks or more and dosage 30-39 mg/day | 2 |
|  |  | 4 wks or more and dosage 40-59 mg/day | 3 |
|  |  | 4 wks or more and dosage 60 mg/day | 4 |
|  | Venlafaxine (Effexor and Effexor XL) | drug < 4 wks or 4 wks or more and dosage < 75 mg/day | 1 |
|  |  | 4 wks or more and dosage 75-224 mg/day | 2 |
|  |  | 4 wks or more and dosage 225-374 mg/day | 3 |
|  |  | 4 wks or more and dosage 375 mg/day | 4 |
| NaSSA | Mirtazapine (Zispin) | drug < 4 wks or 4 wks or more and dosage < 15 mg/day | 1 |
|  |  | 4 wks or more and dosage 15-29 mg/day | 2 |
|  |  | 4 wks or more and dosage 30-44 mg/day | 3 |
|  |  | 4 wks or more and dosage 45 mg/day | 4 |

ATHF: Antidepressant Treatment History Form; SSRI: Selective Serotonin Reuptake Inhibitor; SNRI: Serotonin-Norepinephrine Reuptake Inhibitor; NaSSA: Noradrenergic and Specific Serotonergic Antidepressant

**4.Table S2:** **Non-significant correlation values between clinical symptoms and FCs**

| FCs | HAMD-17 | HAMA |
| --- | --- | --- |
| AMYG.L-PUT.L | *r* = -0.032, *P* = 0.869 | *r* = 0.057, *P* = 0.765 |
| AMYG.R-IFGoperc.R | *r* = 0.203, *P* = 0.282 | *r* = 0.155, *P* = 0.415 |
| AMYG.R-SAM.R | *r* = -0.098, *P* = 0.607 | *r* = 0.279, *P* = 0.135 |
| AMYG.R-DCG.L | *r* = -0.062, *P* = 0.746 | *r* = -0.072, *P* = 0.705 |
| AMYG.R-MOG.L | *r* = 0.050, *P* = 0.793 | *r* = 0.331, *P* = 0.074 |
| AMYG.R-IOG.L | *r* = 0.229, *P* = 0.224 | *-* |
| AMYG.R-PCUN.L | *r* = 0.021, *P* = 0.913 | *r* = 0.096, *P* = 0.614 |
| AMYG.R-PCL.L | *r* = 0.013, *P* = 0.947 | *r* = 0.061, *P* = 0.749 |

AMYG.L: left amygdala; AMYG.R: right amygdala; PUT.L: left putamen; IFGoperc.R: right inferior frontal gyrus; SAM.R: right supplementary motor area; DCG.L: paracingulate gyrus; MOG.L: left middle occipital gyrus; IOG.L: left inferior occipital gyrus; PCUN.L:left precuneus; PCL.L:left paracentral lobule

**Supplementary Legends**

Supplementary Figure S1 | (A) TFR and statistical comparison of amygdala activation during implicit (IMP) and explicit (EXP) modes in major depressive disorder patients. (B) TFR and statistical comparison of amygdala activation during implicit and explicit modes in healthy controls (HC).

Supplementary Figure S2 | TFR and statistical comparison of bilateral amygdala responses to sad versus neutral faces within the MDD group under the implicit condition.

Supplementary Figure S3 | **TFR and statistical comparison of bilateral amygdala responses to neutral face between the MDD and HC groups under the implicit condition.**
