# Supplementary figures and images for: Spatiotemporal dynamics of amygdala during implicit and explicit facial emotional recognition in major depressive disorder: An MEG study
Source: Psychol Med. 2025 Oct 24;55:e318. doi: 10.1017/S0033291725101888 (PMC12558623; doi:10.1017/S0033291725101888)

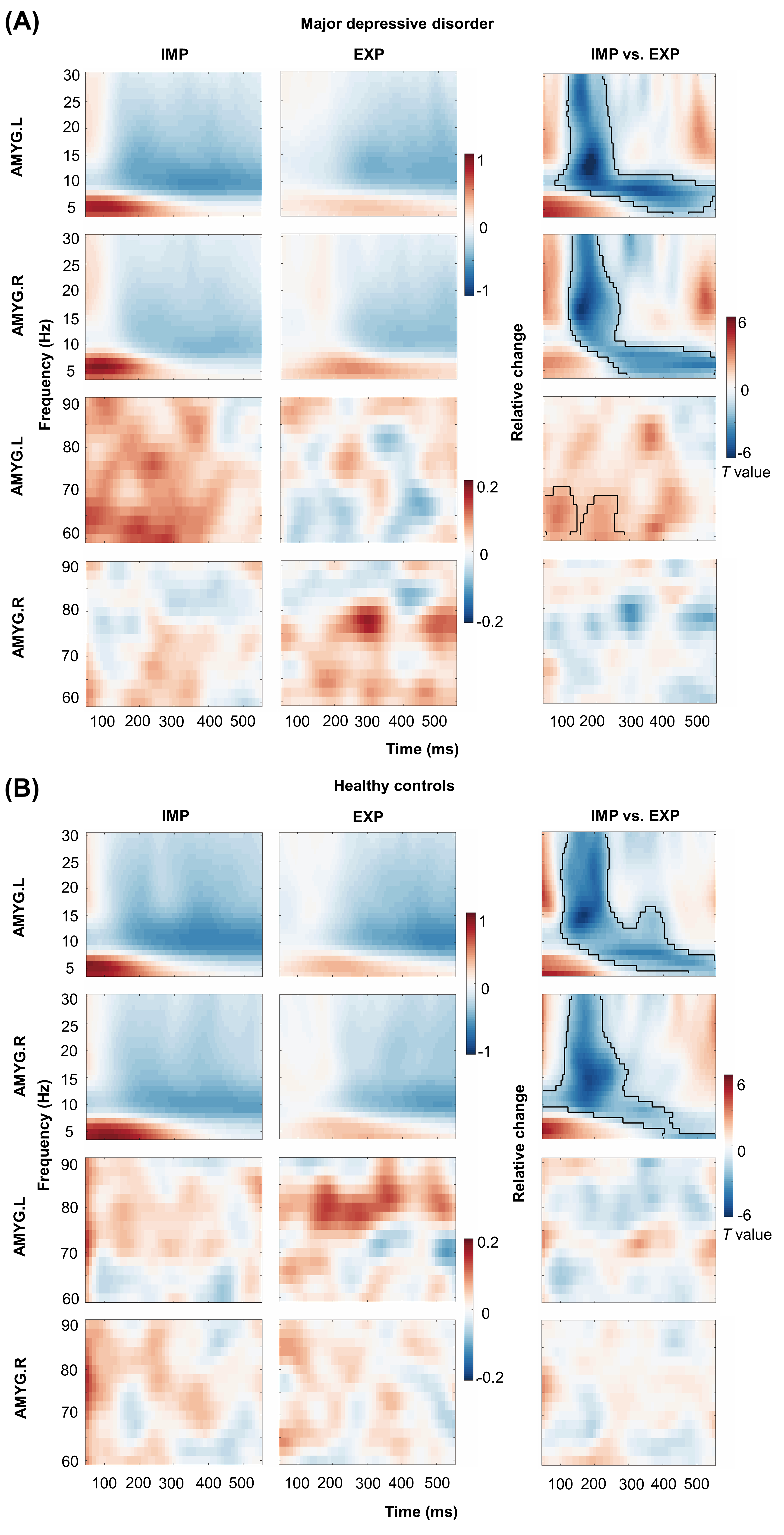

Supplement: Du et al. supplementary material [file S0033291725101888sup001.zip › Supplementary Figure_S1.tiff]

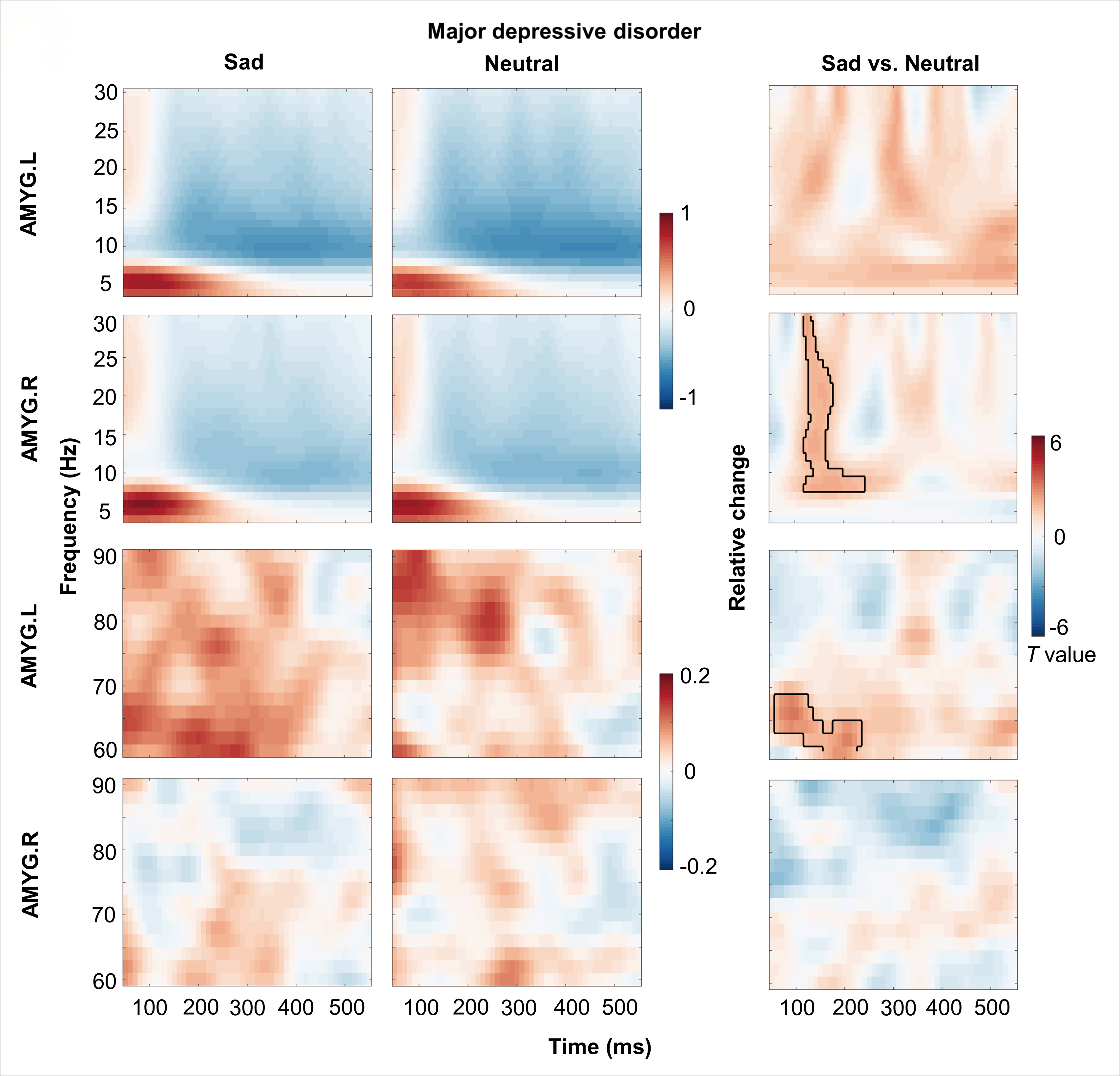

Supplement: Du et al. supplementary material [file S0033291725101888sup001.zip › Supplementary Figure_S2.tiff]

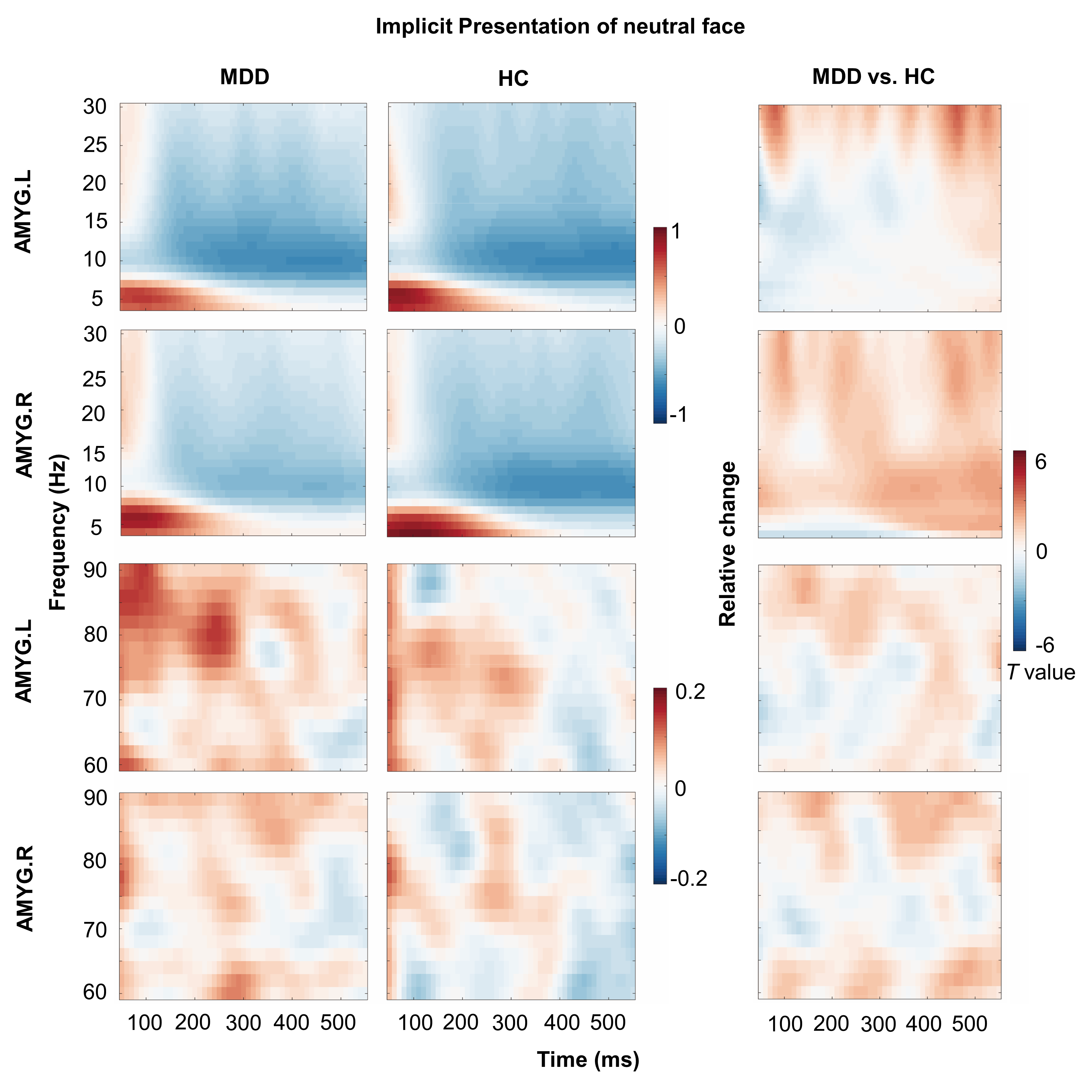

Supplement: Du et al. supplementary material [file S0033291725101888sup001.zip › Supplementary Figure_S3.tiff]
